# Eczema Care Online behavioural interventions to support self-care for children and young people: two independent, pragmatic, randomised controlled trials

**DOI:** 10.1136/bmj-2022-072007

**Published:** 2022-12-08

**Authors:** Miriam Santer, Ingrid Muller, Taeko Becque, Beth Stuart, Julie Hooper, Mary Steele, Sylvia Wilczynska, Tracey H Sach, Matthew J Ridd, Amanda Roberts, Amina Ahmed, Lucy Yardley, Paul Little, Kate Greenwell, Katy Sivyer, Jacqui Nuttall, Gareth Griffiths, Sandra Lawton, Sinéad M Langan, Laura M Howells, Paul Leighton, Hywel C Williams, Kim S Thomas

**Affiliations:** 1 Primary Care Research Centre, Faculty of Medicine, University of Southampton, Southampton SO16 5ST, UK; 2 Centre for Evaluation and Methods, Wolfson Institute of Population Health, Faculty of Medicine and Dentistry, Queen Mary University of London, London, UK; 3 King’s College London, Institute of Psychiatry, Psychology and Neuroscience, London, UK; 4 Health Economics Group, Norwich Medical School, University of East Anglia, Norwich, UK; 5 Population Health Sciences, Bristol Medical School, University of Bristol, Bristol, UK; 6 Centre of Evidence Based Dermatology, School of Medicine, University of Nottingham, Nottingham, UK; 7 School of Psychology, University of Southampton, Southampton, UK; 8 School of Psychological Science, University of Bristol, Bristol, UK; 9 Southampton Clinical Trial Unit, University of Southampton and University Hospital Southampton NHS Foundation Trust, Southampton, UK; 10 The Rotherham NHS Foundation Trust, Rotherham, UK; 11 Department of Non-communicable Disease Epidemiology, London School of Hygiene and Tropical Medicine, London, UK

## Abstract

**Objective:**

To determine the effectiveness of two online behavioural interventions, one for parents and carers and one for young people, to support eczema self-management.

**Design:**

Two independent, pragmatic, parallel group, unmasked, randomised controlled trials.

**Setting:**

98 general practices in England.

**Participants:**

Parents and carers of children (0-12 years) with eczema (trial 1) and young people (13-25 years) with eczema (trial 2), excluding people with inactive or very mild eczema (≤5 on POEM, the Patient-Oriented Eczema Measure).

**Interventions:**

Participants were randomised (1:1) using online software to receive usual eczema care or an online (www.EczemaCareOnline.org.uk) behavioural intervention for eczema plus usual care.

**Main outcome measures:**

Primary outcome was eczema symptoms rated using POEM (range 0-28, with 28 being very severe) every four weeks over 24 weeks. Outcomes were reported by parents or carers for children and by self-report for young people. Secondary outcomes included POEM score every four weeks over 52 weeks, quality of life, eczema control, itch intensity (young people only), patient enablement, treatment use, perceived barriers to treatment use, and intervention use. Analyses were carried out separately for the two trials and according to intention-to-treat principles.

**Results:**

340 parents or carers of children (169 usual care; 171 intervention) and 337 young people (169 usual care; 168 intervention) were randomised. The mean baseline POEM score was 12.8 (standard deviation 5.3) for parents and carers and 15.2 (5.4) for young people. Three young people withdrew from follow-up but did not withdraw their data. All randomised participants were included in the analyses. At 24 weeks, follow-up rates were 91.5% (311/340) for parents or carers and 90.2% (304/337) for young people. After controlling for baseline eczema severity and confounders, compared with usual care groups over 24 weeks, eczema severity improved in the intervention groups: mean difference in POEM score −1.5 (95% confidence interval −2.5 to −0.6; P=0.002) for parents or carers and −1.9 (−3.0 to −0.8; P<0.001) for young people. The number needed to treat to achieve a 2.5 difference in POEM score at 24 weeks was 6 in both trials. Improvements were sustained to 52 weeks in both trials. Enablement showed a statistically significant difference favouring the intervention group in both trials: adjusted mean difference at 24 weeks −0.7 (95% confidence interval −1.0 to −0.4) for parents or carers and −0.9 (−1.3 to −0.6) for young people. No harms were identified in either group.

**Conclusions:**

Two online interventions for self-management of eczema aimed at parents or carers of children with eczema and at young people with eczema provide a useful, sustained benefit in managing eczema severity in children and young people when offered in addition to usual eczema care.

**Trial registration:**

ISRCTN registry ISRCTN79282252.

## Introduction

Atopic eczema, also called atopic dermatitis, and referred to here as eczema^1^ is a common long term condition that can have a substantial impact on the quality of life of both children and adults.^2^ ^3^ Even relatively simple treatment regimens for eczema can be burdensome,^4^ consisting of avoidance of triggers and irritants,^5^ ^6^ regular emollient treatment, and use of topical anti-inflammatory agents such as corticosteroids.

Although eczema guidelines stress the importance of education about eczema,^5^ ^6^ international data suggest that availability of eczema education programmes is sparse in most countries.^7^ Furthermore, systematic reviews have shown limited evidence of benefit for educational, psychological, or self-management interventions in improving eczema outcomes or quality of life.^8^ ^9^ ^10^ One trial showed improved eczema outcomes after group training for eczema involving 12 hours of face-to-face meetings with a multidisciplinary team.^11^ A six hour nurse led education programme for parents of children with eczema, evaluated in a non-randomised study, showed good parental satisfaction and improved eczema from baseline,^8^ ^12^ but 41% of the families who were referred to the programme did not attend,^8^ suggesting barriers to uptake. Implementation of such programmes is resource intensive for patients, families, and health services.

Self-management support for long term health conditions through online interventions has been shown to be associated with small but positive improvements in health outcomes,^13^ particularly theory based interventions that incorporate multiple behaviour change techniques.^14^ Despite the self-management of eczema presenting particular challenges, there have been few rigorously developed online interventions for eczema,^10^ and none have been evaluated in a trial large enough to detect differences in health outcomes.^15^ ^16^ ^17^

We evaluated two online (www.EczemaCareOnline.org.uk; video 1) behavioural interventions to support self-management of eczema: one aimed at the parents or carers of children with eczema, and the other aimed at young people with eczema. As parents and carers of children and young people with eczema are likely to have different support needs, we developed two separate interventions to be evaluated in two independent randomised controlled trials.

## Methods

The Eczema Care Online trials were two separate pragmatic, multicentre, unmasked, individually randomised, superiority trials, each with two parallel groups allocated in a 1:1 ratio comparing usual care alone with an online intervention plus usual care. One trial recruited parents and carers of children aged 0-12 years with eczema and the other recruited young people aged 13-25 years with eczema. The trials were conducted within general practices in the UK National Health Service. The trials included health economic and process evaluations, which will be reported separately. We have previously published the protocol for the trials,^18^ development papers detailing both interventions,^19^ ^20^ and a feasibility trial of a previous prototype intervention.^15^

As described in the published protocol paper,^18^ a protocol amendment was made to revise the sample size in response to new information on the minimal clinically important difference of the primary outcome measure, the Patient-Oriented Eczema Measure (POEM). Our original sample size used a POEM score for minimal clinically important difference of 3, which was based on research carried out in secondary care among people with moderate or severe eczema.^21^ Fresh evidence, however, suggested that a change in POEM score of 2.1 to 2.9 represents a change likely to be beyond measurement error.^22^ A protocol amendment was therefore made to change the target sample size for the trials based on seeking to detect a difference in POEM score of 2.5 points between groups, increasing the target sample size from 200 to 303 for each trial.

### Setting and participants

Participants were invited through a search of electronic health records and postal invitation from participating practices around four regional centres: Wessex, West of England, East Midlands, and Thames Valley and South Midlands. Potential participants were sent an invitation pack containing an information sheet and the study URL to register if they wished to take part. After registering, participants were asked to provide informed consent and complete screening and baseline measures online. For children younger than 16 years, the invitation was sent to their parent or carer. In the trial for parents and carers, informed consent and questionnaires were completed by the parent or carer. In the trial for young people, parental consent and young people’s assent were sought for participants younger than 16 years, and young people’s consent was sought for participants aged 16 and older. Young people aged 13-25 were asked to complete their own questionnaires. 

Eligibility for inclusion in the parents and carers trial included being a parent or carer of a child aged 0-12 years, and eligibility for inclusion in the young people trial included being aged 13-25 years. For both trials, inclusion criteria included child or young individual having a general practice electronic record code for eczema (any date) and having obtained a prescription for eczema treatment (emollient, topical corticosteroid, or topical calcineurin inhibitor) in the 12 months before invitation to the study. On baseline screening for both trials, potential participants were included if a POEM score >5 was reported. This score threshold was used to include those with mild to severe eczema and to exclude those with very mild or inactive eczema to avoid floor effects.^23^

For both trials, potential participants were excluded if they were unable to give informed consent, were unable to read and write English (as the intervention content and outcome measures were in English), had taken part in another eczema study in the past three months, or had no internet access. Only one individual in each household could take part in either trial, as intervention content was similar.

### Interventions

#### Usual care group

Participants randomised to receive usual care were recommended to use a standard informational website,^24^ and they continued to receive usual medical advice and prescriptions from their healthcare provider. They could seek online support but did not have access to Eczema Care Online interventions during their participation in the trial; they were, however, given access to the intervention after the 52 week follow-up.

#### Intervention plus usual care group

Participants randomised to the intervention group received access to Eczema Care Online behavioural interventions in addition to usual eczema care. The interventions were theory based and developed following the person based approach to intervention development,^25^ ^26^ and they were delivered using LifeGuide software. The two interventions were created separately in parallel: one for parents or carers of children with eczema and one for young people with eczema. The interventions were entirely online and self-guided and participants could use as much or as little of the intervention as they wanted. Full details of development and optimisation of both interventions have been published separately.^19^ ^20^ See supplementary appendices 1 and 2 for the TIDieR (template for intervention description and replication) checklists.

The interventions were co-produced by a team consisting of behavioural psychologists, patient representatives, clinicians (general practitioners, dermatology nurse consultants, dermatologists with expertise in eczema) and researchers before being optimised through extensive user feedback to ensure they were acceptable, feasible, and optimally engaging to target users. The aim of the online interventions was to reduce eczema severity and target core behaviours linked to eczema management: regular use of emollients, appropriate use of topical corticosteroids,^27^ avoidance of eczema irritants and triggers, minimisation of scratching, and emotional management.

All intervention content was based on evidence, or on expert consensus when evidence was lacking. The interventions provide tailored content to suggest topics that may be of relevance and include interactive and audio-visual features (eg, brief eczema assessment, videos, stories, and advice from other young people and families with experience of eczema). Participants are taken through a core section comprising key information and behaviour change content about eczema self-management before accessing the main menu with various topics of interest to families and young people with eczema.

### Outcomes

All participant reported outcome measures were collected online using LifeGuide software.^28^ Non-responders were sent reminders by phone or SMS (up to two phone calls or up to two SMS, or both). Outcome measures were similar across the two trials and followed core outcome measures for eczema recommended in the Harmonising Outcome Measures for Eczema international core outcomes set for eczema.^29^ We did not include objective assessment of eczema, however, as this would have required face-to-face contact, which could constitute an intervention in its own right and potentially have greater effect than the online interventions. No changes were made to trial outcomes.

#### Primary outcome

The primary outcome for both trials was the difference in participant reported eczema severity between the usual care group and intervention group, measured by POEM every four weeks over 24 weeks.^23^ ^30^ POEM includes seven questions about the frequency of eczema symptoms over the previous week, with a total score from 0 (no eczema) to 28 (worst possible eczema). POEM can be completed by young people and children or by proxy (parent or carer report) and has good validity, test-retest reliability, and responsiveness to change.^31^ POEM is recommended for measuring the domain of eczema symptoms in the Harmonising Outcomes for Measuring Eczema international core outcome set for eczema.^29^

#### Secondary outcomes

Secondary outcomes included difference in POEM scores every four weeks over 52 weeks; eczema control at 24 and 52 weeks, measured by RECAP (recap for atopic eczema patients)^32^; itch intensity^33^ at 24 and 52 weeks, measured as worst itch in the past 24 hours (not validated for proxy completion for children, and therefore included for young people only); patient enablement at 24 and 52 weeks: the self-perceived ability to understand and cope with health problems, measured using the Patient Enablement Instrument^34^; quality of life at 24 and 52 weeks, measured by proxy using the Child Health Utility-Nine Dimensions (CHU-9D)^35^ for children aged 2-12 years and using the EQ-5D-5L^36^ for young people aged 13-25 (quality of life was not assessed for children aged 0-2 years); and health service use and drug use, measured by review of medical notes for the three month period before baseline and the whole 52 week trial period.

#### Other and process measures

At baseline, participants were asked for their prior belief about the effectiveness of the intervention and their use of other online resources (websites or apps for eczema).

Self-reported barriers to adherence to eczema treatments were measured at 24 and 52 weeks using the Problematic Experiences of Therapy Scale, and frequency of eczema treatment use (treatment adherence) was measured by self-report at 24 and 52 weeks. LifeGuide software recorded the data on intervention usage (eg, time spent on the intervention, number of logins, pages viewed) for each participant for the duration of the 52 week trial period. A full process evaluation is currently in preparation; in this paper we report proportions of users meeting the minimum effective engagement threshold that we predefined for the interventions—that is, completing the core content.^37^ ^38^ Health service use and drug use will be reported separately as part of a full health economic evaluation.

### Sample size

The sample size calculation was based on POEM scores every four weeks using repeated measures from baseline to 24 weeks, seeking to detect a minimum clinically important difference of 2.5 (standard deviation 6.5) points between groups. Assuming a correlation between repeated measures of 0.70, with 90% power and 5% significance, this would give a target sample size of 121 in each group in each of the two trials. Allowing for 20% loss to follow-up resulted in a target sample size of 303 in each of the two trials.

### Randomisation and masking

Participants were randomised online using LifeGuide software either to usual eczema care or to online intervention plus usual care. Randomisation was carried out in random permuted blocks (sizes 4 and 6) and stratified by age (children 0-5 *v* 6-12 years; young people 13-17 *v* 18-25 years), baseline eczema severity (POEM categories^23^ 6-7 (mild), 8-16 (moderate), 17-28 (severe)), and recruitment region (four regions). It was not possible to mask participants to their allocation group, but their prior belief in the effectiveness of the online intervention was measured at baseline to minimise potential bias. The trial management group and statisticians remained blinded to treatment allocation during the conduct of the study and analysis.

### Statistical analysis

Analysis was conducted according to a statistical analysis plan agreed in advance with the independent trial steering committee or data monitoring committee and reported according to CONSORT (consolidated standards of reporting trials) guidelines.^39^ ^40^ The two trials (parents or carers, and young people) were analysed separately. We used descriptive statistics to compare baseline characteristics of trial participants by allocated group. The primary analyses for the total POEM score used generalised linear mixed models with observations over time from week 1 to week 24 (level 1) nested within participants (level 2). Our primary outcome is based on adjusted results, controlling for age, baseline POEM score, recruiting centre, sex, ethnicity, prior belief in the intervention, previous use of a website or app for eczema, and parental education (in the parent and carer trial). We also report unadjusted results for the primary outcome.

Participants who had at least one follow-up POEM score between weeks 6 and 24 were included in the primary repeated measures analysis. For all models, participants were analysed in the group to which they were randomised, regardless of their adherence to that allocation (intention-to-treat analysis).

The model used all the observed data and implicitly assumes that, given the observed data, missing POEM scores were missing at random. The model included a random effect for centre (random intercept) and patient (random intercept and slope on time) to allow for differences between participants and between centres at baseline and differences between participants in the rate of change over time if a treatment-time interaction was statistically significant, and fixed effects for baseline covariates. We initially fitted this model (as specified in the statistical analysis plan), but as the intraclass correlation coefficient for regional centre was <0.001, regional centre was included as a fixed effect (rather than a random effect) in the final model. An unstructured covariance matrix was used. We examined the structure and pattern of missing data, and multiple imputation was performed as a sensitivity analysis. The imputation model included all the covariates in the analysis model, as well as any covariates predictive of missingness. Overall, 100 imputed datasets were generated using multiple imputation with chained equations, and the data was analysed using the same model as for the primary analysis.

For the analysis of secondary outcomes, we used repeated measures analysis for the monthly POEM measure up to 52 weeks consistent with that used for the primary outcome. For other secondary outcomes, linear regression was used for continuous outcomes if the assumptions were met. Logistic regression was used for dichotomous outcomes. When appropriate, we analysed highly skewed variables as dichotomous outcomes. All secondary analyses controlled for baseline value, recruiting centre, age, sex, ethnicity, prior belief in the intervention, previous use of a website or app for eczema, and parental education (in the parent and carer trial). The data were analysed using Stata version 16.

### Patient and public involvement

The James Lind Alliance Priority Setting Partnership for eczema prioritised the most effective form of eczema education as a key research question.^41^ Public contributor AR has been involved in supporting eczema management for many years, including through the internet, and was involved in both the Priority Setting Partnership and in the feasibility trial before the full scale trial reported here. Public contributors AR, AA, and other members of the Centre of Evidence Based Dermatology patient panel were involved from the earliest stages of planning the grant application and subsequently in developing trial recruitment materials and interventions. AR was a member of the trial management group. Public contributors were involved in study interpretation and planning dissemination of findings. The independent trial steering committee included representation from key eczema charities in the UK, also involved in planning dissemination.

## Results

### Participant characteristics

Recruitment took place from 2 December 2019 to 8 December 2020, with follow-up completed in December 2021. Recruitment was paused in April-May 2020 in response to the covid-19 pandemic. General practitioners sent invitations to the parents or carers of 8153 children, and 524 (6.4%) consented online to participate, of whom 340 met eligibility criteria and were randomised. Invitations were sent to 5548 young people (or their parent or carer if younger than 16 years), and 411 (7.4%) consented online to participate, of whom 337 met eligibility criteria and were randomised; three subsequently withdrew from follow-up.

**Fig 1:**
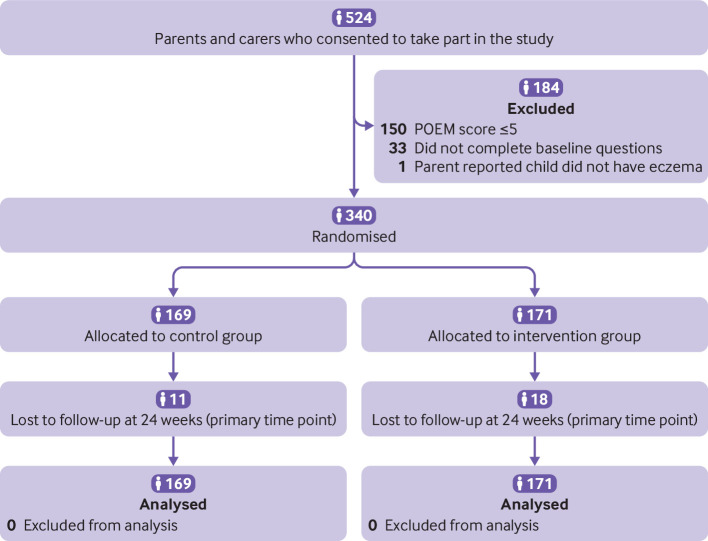
Recruitment of parents or carers of children with eczema

**Fig 2:**
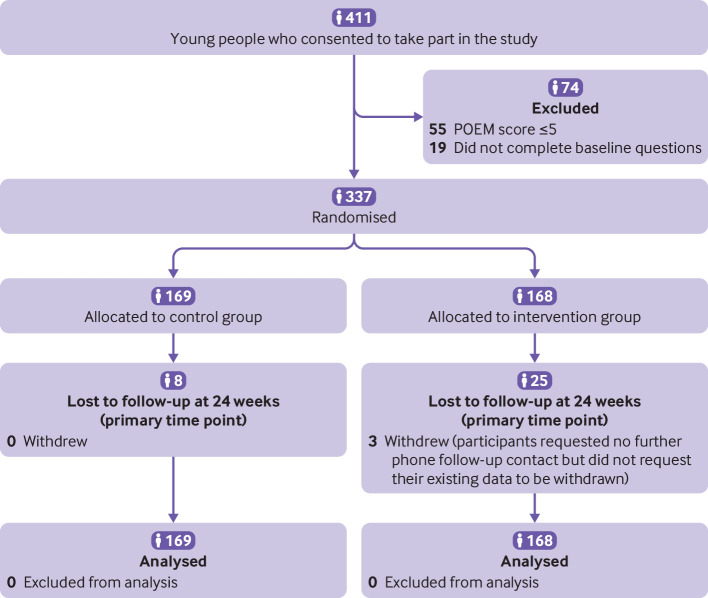
Recruitment of young people with eczema

At 24 weeks (primary time point), POEM was completed by 311/340 (91.5%) parents or carers and 304/337 (90.2%) young people. At 52 weeks, POEM was completed by 303/340 (89.1%) parents or carers and 283/337 (84.0%) young people (fig 1 and fig 2). Participant characteristics in both trials were well balanced at baseline (table 1 and table 2).

**Table 1:** Baseline characteristics of participants in trial for parents or carers of children (0-12 years) with eczema. Values are numbers (percentages) unless stated otherwise

Characteristics	Usual care (n=169)	Online intervention plus usual care (n=171)	Total (n=340)
Mean (SD) respondent’s age (years)	37.5 (6.4)	37.7 (6.8)	37.6 (6.6)
Women	155 (92)	156 (91)	311 (92)
Median (IQR) child’s age (years)	4 (2-7)	4 (2-7)	4 (2-7)
Girls	79 (47)	85 (50)	164 (48)
Respondent’s self-reported ethnic group:			
White	138 (82)	144 (84)	282 (83)
Asian	13 (8)	10 (6)	23 (7)
Black	7 (4)	2 (1)	9 (3)
Mixed	6 (4)	7 (4)	13 (4)
Other	2 (1)	6 (4)	8 (2)
Prefer not to answer	2 (1)	2 (1)	4 (1)
Highest qualification:			
Degree or equivalent	87 (53)	80 (48)	167 (50)
Diploma or equivalent	22 (13)	29 (17)	51 (15)
A level	10 (6)	6 (4)	16 (5)
GSCE or O level	14 (9)	19 (11)	33 (10)
None	3 (2)	5 (3)	8 (2)
Other	24 (15)	23 (14)	47 (14)
Prefer not to answer	4 (2)	6 (4)	10 (3)
Median (IQR) prior belief in intervention score*	7 (5-8.5)	7 (5-8)	7 (5-8)
Use of other websites/apps for eczema in past 6 months	31 (19)	41 (24)	72 (22)
Mean (SD) POEM score†	12.8 (5.4)	12.9 (5.2)	12.8 (5.3)
POEM category:			
Mild (6-7)	25 (15)	28 (16)	53 (16)
Moderate (8-16)	110 (65)	102 (60)	212 (62)
Severe (17-28)	34 (20)	41 (24)	75 (22)
Median (IQR) RECAP score‡	11 (8-16)	12 (9-17)	12 (8-16)
Mean (SD) health related quality of life (CHU-9D)	0.86 (0.10)	0.87 (0.09)	0.87 (0.10)

CHU-9D=Child Health Utility-Nine Dimensions; IQR=interquartile range; POEM=Patient-Oriented Eczema Measure; RECAP=recap for atopic eczema patients; SD=standard deviation.

* Belief that a website might be effective in helping eczema: from 1 (not at all effective) to 10 (very effective).

† Measure of eczema severity: from 0 (low) to 28 (high).^23^ ^30^

‡ Measure of eczema control: from 0 (low) to 28 (high).^32^

**Table 2:** Baseline characteristics of participants in trial for young people (13-25 years) with eczema. Values are numbers (percentages) unless stated otherwise

Characteristics	Usual care (n=169)	Online intervention plus usual care (n=168)	Total (n=337)
Mean (SD) respondent’s age (years)	19.0 (3.3)	19.5 (3.5)	19.3 (3.4)
Female respondents	134 (79)	125 (74)	259 (77)
Respondent’s self-reported ethnic group:			
White	142 (86)	143 (86)	285 (86)
Asian	9 (5)	7 (4)	16 (5)
Black	2 (1)	4 (2)	6 (2)
Mixed	10 (6)	9 (6)	19 (6)
Other	3 (2)	3 (2)	6 (2)
Prefer not to answer	-	-	-
Median (IQR) prior belief in intervention score*	6 (5-8)	6 (5-8)	6 (5-8)
Use of other websites/apps for eczema in past 6 months	24 (14)	26 (16)	50 (15)
Mean (SD) POEM score†	15.3 (5.5)	15.1 (5.3)	15.2 (5.4)
POEM category:			
Mild (6-7)	11 (7)	10 (6)	21 (6)
Moderate (8-16)	92 (54)	92 (55)	184 (55)
Severe (17-28)	66 (39)	66 (39)	132 (39)
Median (IQR) RECAP score‡	13 (8.5-17)	13 (10-16)	13 (9-17)
Median (IQR) itch intensity§	6 (4-7)	6 (4-7)	6 (4-7)
Mean (SD) health related quality of life (EQ-5D-5L)	0.80 (0.18)	0.80 (0.14)	0.80 (0.16)

EQ-5D-5L=five level EuroQol; IQR=interquartile range; POEM=Patient-Oriented Eczema Measure; RECAP=recap for atopic eczema patients; SD=standard deviation.

* Belief that a website might be effective in helping eczema: from 1 (not at all effective) to 10 (very effective).

† Measure of eczema severity: from 0 (low) to 28 (high).^23^ ^30^

‡ Measure of eczema control: from 0 (low) to 28 (high).^32^

§ Measure of itch intensity: from 1 (low) to 10 (high): “How would you rate your itch at the worst moment during the previous 24 hours?” was included for young people only as not validated for use by proxy.

### Primary outcome

#### Trial for parents and carers

**Fig 3:**
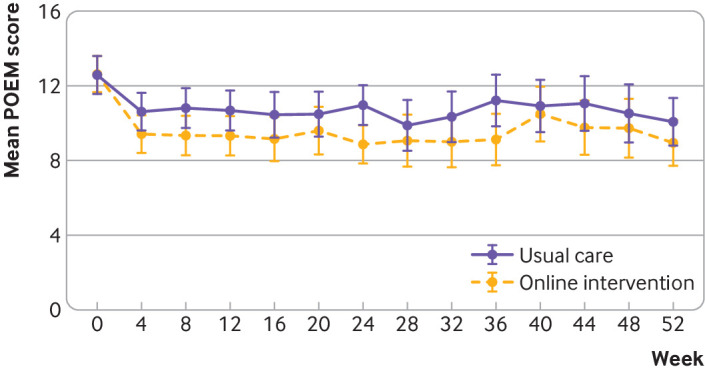
Mean Patient-Oriented Eczema Measure (POEM) scores for eczema severity to 52 weeks in parent and carer trial

Among reports from parents and carers, eczema severity showed improvement by four weeks and appeared relatively constant over time (fig 3). Parent or carer reported mean POEM score for children over the 24 week period was 10.7 in the usual care group and 9.5 in the intervention group. After adjusting for baseline POEM score, recruitment region, age, sex, ethnicity, parental education, prior belief in the intervention, and previous use of a website or app for eczema, the mean difference was −1.5 (95% confidence interval −2.5 to −0.6; P=0.002) between groups, showing a small but statistically significant benefit in POEM scores in the intervention group (table 3). Analysis to assess proportions achieving the minimally important clinical difference of 2.5 points was carried out as a post-hoc analysis to aid interpretation. Overall, 39% (62) of participants in the usual care group and 58% (89) in the intervention group reported an improvement of at least 2.5 points in the POEM score at 24 weeks, giving an odds ratio of 2.1 (95% confidence interval 1.2 to 3.6) corresponding to a number needed to treat of 6 (95% confidence interval 3 to 13).

**Table 3:** Primary outcome: POEM scores over 24 weeks (repeated measures analysis) in trial for parents and carers of children (0-12 years) with eczema

Follow-up	Mean POEM score			Mean difference in score (95% CI)		
	Usual care (n=169)	Online intervention plus usual care (n=171)		Unadjusted	Adjusted*	Adjusted†
24 weeks	10.7	9.5		−1.1 (−2.2 to 0.04)	−1.1 (−2.0 to −0.3)	−1.5 (−2.5 to −0.6)***

CI=confidence interval; POEM=Patient-Oriented Eczema Measure.

* Adjusted for stratification factors: baseline POEM score, recruitment region, and age.

† Adjusted for baseline POEM score, recruitment region, age, sex, ethnicity, parental education, prior belief in the intervention, and previous use of a website/app for eczema.

*** P=0.002.

#### Trial for young people

**Fig 4:**
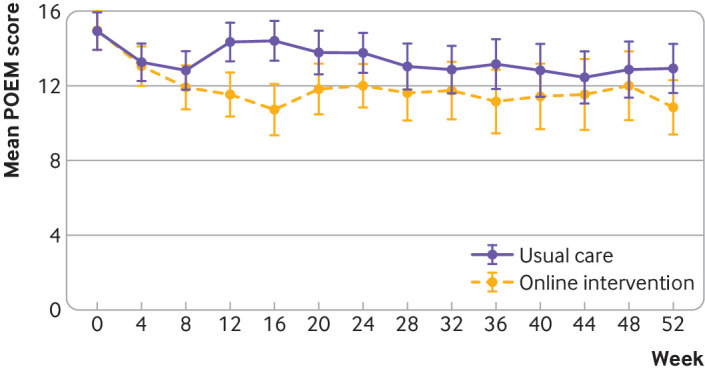
Mean Patient-Oriented Eczema Measure (POEM) scores for eczema severity to 52 weeks in young people trial

Among young people, the mean POEM score over 24 weeks was statistically significant for the treatment-time interaction (P=0.006) showing that improvement developed over several weeks. As the treatment effect varied significantly over the first 24 weeks, scores for each time point are reported (table 4). After adjusting for baseline POEM score, recruitment region, age, sex, ethnicity, prior belief in the intervention, and previous use of a website or app for eczema, the mean difference in POEM score over 24 weeks was −1.9 (95% confidence interval −3.0 to −0.8; P<0.001) between groups, showing a small but statistically significant benefit in POEM scores in the intervention group (fig 4 and table 4). Overall, 39% (63) of participants in the usual care group and 56% (80) in the intervention group reported an improvement of at least 2.5 points in the POEM score at 24 weeks, giving an odds ratio 2.0 (95% confidence interval 1.2 to 3.5) corresponding to a number needed to treat of 6 (95% confidence interval 4 to 18).

**Table 4:** Primary outcome: POEM scores over 24 weeks (repeated measures analysis) in trial for young people (13-25 years) with eczema

Follow-up	Usual care (n=169)			Online intervention plus usual care (n=168)			Mean difference in score (95% CI)		
	No	Mean POEM score		No	Mean POEM score		Unadjusted	Adjusted*	Adjusted†
Week 4	161	13.6		158	12.9		−0.7 (−2.0 to 0.6)	−0.6 (−1.6 to 0.5)	−0.1 (−1.3 to 1.0)
Week 8	139	13.2		119	12.1		−1.1 (−2.5 to 0.3)	−0.9 (−2.1 to 0.3)	−1.0 (−2.3 to 0.4)
Week 12	135	14.4		115	11.6		−2.7 (−4.1 to −1.3)	−2.6 (−3.8 to −1.4)	−2.7 (−4.1 to −1.4)
Week 16	122	14.3		75	11.2		−3.2 (−4.7 to −1.6)	−2.9 (−4.3 to −1.5)	−3.8 (−5.4 to −2.2)
Week 20	103	13.8		74	11.5		−2.3 (−3.9 to −0.6)	−2.1 (−3.7 to −0.6)	−2.1 (−3.8 to −0.4)
Week 24	161	13.9		143	11.8		−2.1 (−3.6 to −0.5)	−1.9 (−3.3 to −0.5)	−1.7 (−3.3 to −0.1)
Over 24 weeks		13.8			11.9		−2.0 (−3.2 to −0.8)	−1.8 (−2.8 to −0.9)	−1.9 (−3.0 to -0.8)***

CI=confidence interval; POEM=Patient-Oriented Eczema Measure.

* Adjusted for stratification factors: baseline POEM score, recruitment region, and age.

† Adjusted for baseline POEM score, recruitment region, age, sex, ethnicity, prior belief in the intervention, and previous use of a website/app for eczema.

*** P<0.001.

Sensitivity analyses using multiply imputed data for missing outcomes showed similar results for both interventions (see appendix tables S3 and S4).

### Secondary outcomes

POEM scores over 52 weeks showed a persisting benefit for the intervention group, with adjusted mean difference in score of −1.4 (95% confidence interval −2.3 to −0.4) in the trial for parents and carers and −1.4 (−2.4 to −0.4) in the trial for young people (fig 3 and fig 4). In the trial for parents and carers, 48% (74) of participants in the usual care group and 60% (89) in the intervention group reported an improvement of at least 2.5 points in the POEM score at 52 weeks (adjusted odds ratio 1.4, 95% confidence interval 0.8 to 2.4). In the trial for young people, 47% (70) of participants in the usual care group and 62% (84) in the intervention group reported an improvement of at least 2.5 points in the POEM score at 52 weeks (adjusted odds ratio 1.6, 0.9 to 2.8).

The only significant difference between groups in secondary outcomes was in the Patient Enablement Instrument, which showed improvements of about 1 point on the 7 point scale in the intervention groups in both trials by 24 weeks (table 5 and table 6): equivalent to a difference from participants in usual care group feeling neutral about being helped to manage their eczema to participants in the intervention group reporting that they were now better able to understand, cope with, and manage their eczema. This difference persisted to 52 weeks in both trials (table 5 and table 6).

**Table 5:** Secondary outcomes in trial for parents and carers of children (0-12 years) with eczema. Values are mean (standard deviation) scores unless stated otherwise

Outcome	No	Usual care (n=169)	No	Online intervention plus usual care (n=171)	Mean difference (95% CI)		
					Unadjusted	Adjusted*	Adjusted†
Eczema severity (POEM) over 52 weeks		10.0 (6.6)		8.9 (6.7)	−1.0 (−2.1 to 0.1)	−1.1 (−1.9 to −0.3)	−1.4 (−2.3 to −0.4)***
Eczema control (RECAP)‡:							
Week 24	121	9.7 (6.3)	116	9.0 (6.1)	−0.7 (−2.3 to 0.9)	−1.0 (−2.4 to 0.4)	−0.6 (−2.3 to 1.0)
Week 52	119	9.4 (6.9)	117	8.6 (6.0)	−0.8 (−2.5 to 0.9)	−0.6 (−2.1 to 1.0)	−0.4 (−2.2 to 1.4)
Patient Enablement Instrument§:							
Week 24	144	3.3 (1.4)	135	2.6 (1.2)	−0.7 (−1.0 to −0.4)	−0.7 (−1.0 to −0.4)	−0.7 (−1.0 to −0.4)***
Week 52	146	3.4 (1.5)	139	2.6 (1.3)	−0.8 (−1.1 to −0.5)	−0.8 (−1.1 to −0.5)	−0.8 (−1.2 to −0.5)***
Health related quality of life (CHU-9D):							
Week 24	126	0.89 (0.10)	122	0.90 (0.09)	0.01 (−0.01 to 0.04)	0.02 (−0.01 to 0.04)	0.01 (−0.02 to 0.03)
Week 52	122	0.88 (0.10)	116	0.90 (0.09)	0.02 (−0.01 to 0.04)	0.02 (−0.01 to 0.04)	0.01 (−0.02 to 0.04)

CHU-9D=Child Health Utility-Nine Dimensions; CI=confidence interval; POEM=Patient-Oriented Eczema measure; RECAP=recap for atopic eczema patients.

* Adjusted for stratification factors: baseline POEM score, recruitment region, and age.

† Adjusted for baseline score, recruitment region, age, sex, ethnicity, parental education, prior belief in the intervention, and previous use of a website/app for eczema.

‡ Measure of eczema control: scores from 0 (low) to 28 (high).^32^

§ Measures self-perceived ability to understand and cope with health problems. Instrument is scored as an average across six questions (I am able to cope better, I am able to understand my eczema better, etc) on a scale 1=strongly agree, 2=agree, 3=slightly agree, 4=neutral, 5=slightly disagree, 6=disagree, 7=strongly disagree.

*** P<0.05.

**Table 6:** Secondary outcomes in trial for young people (13-25 years) with eczema. Values are mean (standard deviation) scores unless stated otherwise

Outcome	No	Usual care (n=169)	No	Online intervention plus usual care (n=168)	Mean difference (95% CI)		
					Unadjusted	Adjusted*	Adjusted†
Eczema severity (POEM) over 52 weeks		12.7 (6.8)		10.7 (6.6)	−1.7 (−2.8 to −0.5)	−1.5 (−2.4 to −0.6)	−1.4 (−2.4 to −0.4)***
Eczema control (RECAP)‡:							
Week 24	133	11.5 (6.3)	109	10.3 (6.0)	−1.2 (−2.8 to −0.4)	−0.9 (−2.4 to 0.5)	−0.2 (−1.6 to 1.6)
Week 52	130	10.7 (6.6)	102	9.2 (6.0)	−1.5 (−3.2 to 0.1)	−1.4 (−3.0 to 0.2)	−1.1 (−3.0 to 0.8)
Itch intensity							
Week 24	160	5.0 (2.5)	139	5.0 (2.6)	0.01 (−0.6 to 0.6)	0.04 (−0.5 to 0.6)	0.3 (−0.3 to 0.9)
Week 52	144	4.7 (2.7)	130	4.5 (2.6)	−0.3 (−0.9 to 0.4)	−0.3 (−0.9 to 0.3)	−0.4 (−1.1 to −0.3)
Patient Enablement Instrument§:							
Week 24	135	3.7 (1.4)	122	2.8 (1.1)	−0.9 (−1.2 to −0.6)	−0.9 (−1.2 to −0.6)	−0.9 (−1.3 to −0.6)***
Week 52	137	3.7 (1.3)	121	2.7 (1.0)	−1.0 (−1.3 to −0.7)	−1.0 (−1.3 to −0.7)	−1.2 (−1.5 to −0.8)***
Health related quality of life (EQ5D-5L):							
Week 24	154	0.80 (0.18)	138	0.80 (0.18)	0.01 (−0.03 to 0.05)	0.01 (−0.04 to 0.05)	0.01 (−0.03 to 0.05)
Week 52	147	0.79 (0.17)	133	0.83 (0.17)	0.03 (−0.01 to 0.07)	0.03 (−0.01 to 0.07)	0.03 (−0.01 to 0.08)

EQ-5D-5L=five level EuroQol; CI=confidence interval; POEM=Patient-Oriented Eczema measure; RECAP=recap for atopic eczema patients.

* Adjusted for stratification factors: baseline POEM score, recruitment region, and age.

† Adjusted for baseline score, recruitment region, age, sex, ethnicity, prior belief in the intervention, and previous use of a website/app for eczema.

‡ Measure of eczema control: scores from 0 (low) to 28 (high).^32^

§ Measures self-perceived ability to understand and cope with health problems. Instrument is scored as an average across six questions (I am able to cope better, I am able to understand my eczema better, etc) on a scale 1=strongly agree, 2=agree, 3=slightly agree, 4=neutral, 5=slightly disagree, 6=disagree, 7=strongly disagree.

*** P<0.05.

Other outcomes did not differ between the groups, including in the Problematic Experiences of Therapy Scale, although in the parent and carer trial the perception of treatments as problematic seemed to be lower in the intervention group, although not statistically significant.

Treatment use was highly skewed and was therefore analysed as a dichotomous variable (7 days versus *<*7 days for emollient use, and any versus none for topical corticosteroid and topical calcineurin inhibitor use). No significant differences were found between groups in either trial on any of the measures of treatment use (emollient, topical corticosteroid, topical calcineurin inhibitor) measured at 24 weeks (table 7 and table 8).

**Table 7:** Treatment adherence outcomes in trial for parents and carers of children (0-12 years) with eczema. Values are numbers (percentages) unless stated otherwise

Outcome	Usual care (n=169)	Online intervention plus usual care (n=171)	Odds ratio (95% CI)		
			Unadjusted	Adjusted*	Adjusted†
**Problematic Experiences of Therapy Scale**					
Week 24:					
Symptoms too severe or aggravated by treatment	67 (45)	52 (37)	0.7 (0.5 to 1.2)	0.7 (0.4 to 1.1)	0.6 (0.3 to 1.0)
Uncertainty about how to carry out treatment	48 (32)	40 (28)	0.8 (0.5 to 1.4)	0.8 (0.5 to 1.3)	0.7 (0.4 to 1.3)
Doubts about treatment efficacy	71 (48)	61 (44)	0.8 (0.5 to 1.3)	0.8 (0.5 to 1.3)	0.6 (0.3 to 1.1)
Practical problems	77 (53)	78 (57)	1.2 (0.7 to 1.8)	1.1 (0.7 to 1.8)	0.9 (0.5 to 1.6)
Week 52:					
Symptoms too severe or aggravated by treatment	61 (42)	54 (39)	0.9 (0.5 to 1.4)	0.9 (0.5 to 1.4)	1.0 (0.5 to 1.7)
Uncertainty about how to carry out treatment	44 (31)	40 (28)	0.9 (0.5 to 1.5)	1.0 (0.6 to 1.6)	0.8 (0.4 to 1.5)
Doubts about treatment efficacy	67 (47)	52 (37)	0.7 (0.4 to 1.1)	0.6 (0.4 to 1.0)	0.5 (0.3 to 0.9)***
Practical problems	76 (54)	79 (57)	1.1 (0.7 to 1.8)	1.2 (0.7 to 1.9)	0.9 (0.5 to 1.5)
**Treatment use**					
Week 24:					
Emollients:					
0-6 days/wk	59 (38)	51 (35)	-		-
7 days/wk	95 (62)	97 (66)	1.2 (0.7 to 1.9)	1.2 (0.7 to 1.9)	1.4 (0.7 to 2.5)
Topical: corticosteroid or calcineurin inhibitor:					
0 days/wk	72 (46)	54 (36)	-		-
1-7 days/wk	83 (54)	94 (64)	1.5 (1.0 to 2.4)	1.6 (1.0 to 2.5)	1.5 (0.8 to 2.8)
Week 52:					
Emollients:					
0-6 days/wk	56 (39)	43 (30)	-		-
7 days/wk	86 (61)	100 (70)	1.5 (0.9 to 2.5)	1.7 (1.0 to 2.8)	2.3 (1.2 to 4.5)***
Topical: corticosteroid or calcineurin inhibitor:					
0 days/wk	66 (47)	58 (41)	-		-
1-7 days/wk	76 (54)	84 (59)	1.3 (0.8 to 2.0)	1.4 (0.8 to 2.2)	1.5 (0.8 to 2.7)

CI=confidence interval.

* Adjusted for stratification factors: baseline POEM score, recruitment region, and age.

† Adjusted for baseline score, recruitment region, age, sex, ethnicity, parental education, prior belief in the intervention, and previous use of a website/app for eczema.

*** P<0.05.

**Table 8:** Treatment adherence outcomes in trial for young people (13-25 years) with eczema. Values are numbers (percentages) unless stated otherwise

Outcome	Usual care (n=169)	Online intervention plus usual care (n=168)	Odds ratio (95% CI)		
			Unadjusted	Adjusted*	Adjusted†
**Problematic Experiences of Therapy Scale**					
Week 24:					
Symptoms too severe or aggravated by treatment	85 (56)	76 (57)	1.0 (0.6 to 1.6)	1.0 (0.6 to 1.7)	1.0 (0.6 to 1.9)
Uncertainty about how to carry out treatment	63 (42)	54 (40)	1.0 (0.6 to 1.5)	0.9 (0.6 to 1.5)	1.1 (0.6 to 2.0)
Doubts about treatment efficacy	103 (68)	89 (67)	1.0 (0.6 to 1.6)	1.0 (0.6 to 1.6)	1.1 (0.6 to 2.1)
Practical problems	116 (78)	104 (79)	1.0 (0.6 to 1.8)	1.0 (0.6 to 1.8)	1.1 (0.5 to 2.3)
Week 52:					
Symptoms too severe or aggravated by treatment	80 (55)	71 (55)	1.0 (0.6 to 1.6)	1.0 (0.6 to 1.6)	1.1 (0.6 to 2.0)
Uncertainty about how to carry out treatment	58 (40)	57 (44)	1.2 (0.7 to 1.9)	1.2 (0.7 to 1.9)	1.5 (0.8 to 2.7)
Doubts about treatment efficacy	88 (63)	80 (62)	1.0 (0.6 to 1.6)	1.0 (0.6 to 1.6)	0.9 (0.5 to 1.7)
Practical problems	116 (81)	111 (85)	1.4 (0.8 to 2.7)	1.5 (0.8 to 2.8)	1.4 (0.6 to 3.1)
**Treatment use**					
Week 24:					
Emollient:					
0-6 days/wk	71 (44)	64 (46)	-		-
7 days/wk	89 (56)	74 (54)	0.9 (0.6 to 1.5)	0.9 (0.6 to 1.5)	1.2 (0.7 to 2.2)
Topical: corticosteroid or calcineurin inhibitor:					
0 days/wk	61 (38)	55 (40)	-		-
1-7 days/wk	98 (62)	83 (60)	0.9 (0.6 to 1.6)	0.9 (0.6 to 1.5)	1.0 (0.5 to 1.8)
Week 52:					
Emollient:					
0-6 day/wk	66 (46)	60 (46)	-		-
7 days/wk	79 (55)	72 (55)	1.0 (0.6 to 1.6)	1.0 (0.6 to 1.6)	0.9 (0.5 to 1.8)
Topical: corticosteroid or calcineurin inhibitor:					
0 days/wk	51 (35)	48 (36)	-		-
1-7 days/wk	93 (65)	84 (64)	1.0 (0.6 to 1.6)	0.9 (0.6 to 1.5)	0.9 (0.5 to 1.7)

CI=confidence interval.

* Adjusted for stratification factors: baseline POEM score, recruitment region, and age.

† Adjusted for baseline score, recruitment region, age, sex, ethnicity, prior belief in the intervention, and previous use of a website/app for eczema.

Analysis of completion of core content (predefined minimum effective engagement threshold) was excellent: data for online intervention usage showed that most participants had completed the core module by 24 weeks: 299/340 (88%) parents and carers and 310/337 (92%) young people.

### Subgroup analyses

Prespecified subgroup analyses in both trials showed that participants allocated to the intervention group showed similar benefit in eczema outcomes, regardless of age, sex, eczema severity, baseline treatment use, prior belief in effectiveness of intervention, or previous use of other eczema related websites (see appendix tables S5 and S6). No harms or unintended effects were identified in either trial.

## Discussion

This study found that two brief online behavioural interventions to enable self-management of eczema for parents and carers of children with eczema and for young people with eczema provided a useful benefit in eczema severity at 24 weeks, which was sustained at 52 weeks. A number needed to treat of 6 compares favourably with many drug treatments and is particularly important in the absence of identifiable harms and in the context of a low cost and highly scalable intervention.

Use of eczema treatments did not differ between groups, but scores on the Patient Enablement Instrument differed significantly. We therefore believe that the impact of the interventions may have been through enabling parents and carers of children with eczema and young people with eczema to feel more confident in coping with the condition. The process evaluation will be reported separately and will provide insights into the mechanism of action of the interventions.

The Eczema Care Online toolkits were offered to the intervention group in addition to usual eczema care. The toolkits therefore should be viewed as supplementing rather than replacing health professional support.

### Strengths and limitations of this study

The two Eczema Care Online trials have several strengths, including long follow-up, high rates of follow-up, broad inclusion criteria and range of eczema severities, and outcome measures of importance to young people with eczema and carers, leading overall to a pragmatic trial and generalisable results.

It was not possible to blind participants to treatment allocation, and this could have led to bias in the primary outcome, despite measures to adjust for prior belief in the intervention to minimise this potential bias in analysis. However, even if a contextual effect (or placebo effect) contributes to improvement in eczema, the effect is still a valuable benefit to people with eczema and their families, particularly when it improves their ability to cope with the condition.

The improvements in primary outcome (1.5 (95% confidence interval 0.6 to 2.5) for children and 1.9 (0.8 to 3.0) for young people) were less than the target of 2.5 points on the POEM score. The most recent research on the minimal clinically important difference for POEM suggests that a range of 2.1 to 2.9 represents a small change that is likely to be beyond measurement error, and that a “small improvement in many individuals could result in a large reduction in burden at a societal level.”^22^ Our estimates fall below this but with narrow confidence intervals that exclude the null hypothesis and include the minimal clinically important difference. However, substantial proportions of participants experienced clinically important improvement: more than half in the intervention group in both trials achieved an improvement at or above the minimal clinically important difference, and the number needed to treat for one participant to benefit compared with usual care was 6 in both trials, which is noteworthy for such a low cost intervention.

Recruitment into this trial was through a search of general practice records and postal invitations to potentially eligible participants. Although this method for recruitment resulted in a low response rate, it is consistent with other similar studies,^42^ which means that the invitation to participate will not always be salient to people because eczema is a relapsing-remitting condition and people are unlikely to respond when in remission. In real world use, the interventions are envisaged as being particularly appropriate around newly diagnosed eczema or flare-ups, where uptake is likely to be higher and the intervention could potentially be most effective.

Some of the recruitment and follow-up of participants in this study took place during the covid-19 pandemic. Qualitative research carried out during the trial suggested that this could have had both positive and negative impacts on participants’ eczema.^43^ For example, it may have made it harder for participants to access healthcare for some months during the study and to discuss or change their treatments in response to the intervention, although this lack of access may have improved engagement with the online toolkits.

### Comparison with other studies

Few fully powered trials have been carried out of self-management or educational interventions for eczema, and those that have been published used different outcome measures, making direct comparisons challenging. However, much more costly educational interventions have only shown modest improvements in eczema, and we believe the effect size in our trials compares favourably with more intensive interventions.

In some contexts, the effectiveness of online interventions has been shown to be enhanced by health professional support, and this was tested in a feasibility study before this trial.^15^ In the three arm feasibility study, 143 parents or carers of children with eczema were randomised to: usual care alone; an online intervention plus usual care; or an online intervention plus 20 minutes of health professional support (primarily practice nurses) and usual care. In the feasibility study, health professional support did not lead to better outcomes, and process evaluation indicated that the health professional support was not highly valued by participants in this context, and it was therefore not included in the full scale trial reported here.

### Implications for practice and future research

As 90% of people with eczema are managed in primary care in the UK, further research is needed to explore the impact of online interventions in healthcare settings where secondary care management is more common or where patient support for eczema is more extensive. Although some aspects of the Eczema Care Online interventions are specific to the UK, such as available treatments and support for navigating health services, the intervention could readily be adapted to other settings.

### Conclusions

Eczema Care Online interventions for parents and carers of children with eczema and for young people with eczema are evidence based resources that have been shown to help young people better understand, cope with, and manage their eczema, and offer a useful benefit in clinical outcomes, sustained over 52 weeks. A small amount of benefit at low cost with no identifiable harms for a condition that affects a large number of people can lead to substantial health benefit for the public in absolute terms. The findings reinforce the key role of health professionals in signposting patients and carers towards self-management support for long term conditions.

**What is already known on this topic**

People with eczema and their families often report they have been given insufficient or conflicting information about the condition or how to manage it
Group education delivered by multidisciplinary teams has been shown to improve eczema outcomes but is expensive and time consuming to deliver
The effectiveness of online self-management support for eczema has not been assessed in adequately powered trials

**What this study adds**

Online interventions providing evidence based support for eczema self-management led to a useful, sustained benefit in eczema severity over six and 12 months in children and young people
This small but meaningful improvement is particularly valuable given the low cost and high scalability of the online support and absence of identifiable harms

## Data Availability

Consent was not obtained from participants for data sharing. Authors will consider reasonable request to make relevant anonymised participant level data available.

## Web extra

Extra material supplied by authors

Supplementary information: additional material and a video discussing the study findings

## Notes

Contributors: MSa and KST conceived the study and initial study design in collaboration with IM, LY, PLi, HCW, JRC, MJR, SaL, BS, GG, TS, SiL, AR, and AA, with later input from JN, JH, SW, MSt, KG, KS, and TB. Specific advice was given by BS and TB on trial design and medical statistics; IM, LY, KG, KS, PLe, and LH on the process evaluation; and TS on the health economic evaluation. TB and BS conducted the analyses. All the authors contributed to the drafting of this paper, led by MS, and approved the final manuscript. MSa is guarantor. The corresponding author attests that all listed authors meet authorship criteria and that no others meeting the criteria have been omitted.

The lead author (MSa) affirms that the manuscript is an honest, accurate, and transparent account of the study being reported; that no important aspects of the study have been omitted; and that any discrepancies from the study as originally planned and registered have been explained.

Dissemination to participants and related patient and public communities: We have shared our findings with relevant advocacy groups, participants in the trial, their families, and the practices involved. Blogs, a Twitter account, and plain English summaries of papers of related research can be viewed at https://www.nottingham.ac.uk/eco/.

Provenance and peer review: Not commissioned; externally peer reviewed.
