# Supplementary material for: Eczema Care Online behavioural interventions to support self-care for children and young people: two independent, pragmatic, randomised controlled trials
Source: BMJ. 2022 Dec 8;379:e072007. doi: 10.1136/bmj-2022-072007 (PMC11778922; doi:10.1136/bmj-2022-072007)
Supplement: Supplementary file 1 — Supplementary information: additional material and a video discussing the study findings [file sanm072007.ww.pdf]

Full description of Eczema Care Online for Parents/Carers in accordance with the Template for Intervention Description and Replication (TIDieR) checklist

Reproduced from Sivyer K, Teasdale E, Greenwell K, et al. Supporting families managing childhood eczema: Developing and optimising Eczema Care Online using qualitative research. British Journal of General Practice 2022

1. BRIEF NAME: Eczema Care Online for Parents/Carers

2. WHY: Online interventions could potentially be used to support parents/cares in managing their child's eczema; however, research developing such interventions has been limited and systematic reviews show the need for systematically developed evidence-based interventions to support eczema self-management.

3 & 4. WHAT: Eczema Care Online for Parents/Carers is an online behaviour change intervention for parents/carers who have a child aged 0-12 years with mild to severe eczema. It aims to reduce eczema severity through supporting parental management and parent/child co-management of eczema, targeting 5 key behaviours: 1) reactive application of topical corticosteroids (TCS) to get control of skin inflammation; 2) increased use of emollients to keep control of skin inflammation; 3) improved management of irritants / triggers; 4) reduced scratching (in children); 5) improved emotional distress management (in children). See Table S1 for an overview of the modules included in Eczema Care Online for Parents/Carers.

Table S1: Overview of the key intervention components of Eczema Care Online for Parents/Carers

Menu	Modules	Description
Home Page	N/A	• Signposting to key modules that parents/carers haven't accessed yet<br>• Quick links to interactive content e.g. videos
Flare control creams	• Flare control cream video<br>• What are they?<br>• Are they safe?<br>• Common questions<br>• When and how do I use them?<br>• How can I find the right cream?<br>• Golden rules	• Evidence-based education and messages to increase motivation and competence to use topical corticosteroids (TCS), including; evidence of safety and support for skills development e.g. videos/photos showing use of TCS
Moisturising creams	• Moisturising creams video<br>• What are they and how do they help?<br>• Are they safe?<br>• Common Concerns<br>• When and how do I use them?<br>• How do I find the right cream?<br>• Take the two-week challenge!<br>• Golden rules	• Evidence-based education and messages to increase motivation and competence to use emollients, including; evidence of safety and support for skills development e.g. videos/photos showing how to use emollients
What can make eczema worse	• Living with eczema video<br>• Bathing, showering, and washing clothes<br>• Diet and allergies<br>• Weather and holidays	• Evidence-based education and messages to increase motivation and competence in identifying key eczema triggers and managing them, including practical tips

	• Swimming and physical activity	
Help your child manage eczema	• Managing eczema at nursery/school<br>• Making treatment times easier<br>• Teaching your child how to care for their eczema<br>• Preparing for the teen years	• Evidence-based education and messages to increase motivation and competence of parents/carers to involve their child in their treatment and support the transition towards co-management and child self-management, including age-appropriate videos to share with their child
Itch, stress and sleep	• Beat the itch<br>• Sleep<br>• Managing stress for parents<br>• Stress and your child	• Evidence-based education and messages to increase motivation and competence to manage some of the psychosocial impacts of eczema e.g. itching and scratching, disturbed sleep, parental and child stress, including techniques to manage them
More about treatments	• Talking to health professionals<br>• Infections<br>• Topical Calcineurin Inhibitors (TCIs)<br>• Other treatments<br>• Other resources	• Evidence-based education and messages to increase motivation and competence to manage other aspects of treatments e.g. consulting with health professionals and other treatments

5. WHO PROVIDED: Eczema Care Online for Parents/Carers was provided direct to patients via their GP surgeries in this trial.

6. HOW: Eczema Care Online for Parents/Carers is entirely online, although users are encouraged to contact their GP, pharmacist or nurse, for example, to obtain or change their treatments, or to treat potential infections.

7. WHERE: Eczema Care Online for Parents/Carers is intended for any parent/carer who has a child aged 0 to 12 years with mild to severe eczema. It can be implemented in primary or secondary care and can also be used stand-alone. The intervention is online and designed to be accessed using computers, tablets and phones via a web-browser.

8. WHEN and HOW MUCH: In this study, parents/carers were offered access to the intervention if their GP identified they had a child aged 0 to 12 years who had eczema and had received a prescription for this in the previous 12 months. Once registered parents/carers could use the online intervention as much or as little as they liked.

9. TAILORING: Parents/carers had access to all the modules on Eczema Care Online, however the website uses signposting based on four questions about the current state of their child's eczema. Users are then recommended either the module on emollients or flare control creams module depending on whether it appears their child is currently experiencing an eczema flare-up. Key modules can also be recommended, one or two at a time, on the intervention home page if they haven't been viewed yet. These modules/menus are; 1) emollients and flare control creams, 2) diet and allergy, 3) beat the itch 4) making treatment times easier

10. MODIFICATIONS: An additional email was circulated in response to COVID-19 that provided further advice about handwashing.

11. HOW WELL: The intervention has been evaluated in a randomised trial.

Full description of Eczema Care Online for Young People in accordance with the Template for Intervention Description and Replication (TIDieR) checklist

Reproduced from Greenwell K, Ghio D, Sivyer K, et al. Eczema Care Online: development and qualitative optimisation of an online behavioural intervention to support self-management in young people with eczema. BMJ Open 2022;12(4):e056867. doi: 10.1136/bmjopen-2021-056867

1. BRIEF NAME: Eczema Care Online for Young People

2. WHY: Online interventions could potentially be used to support young people with eczema; however, research developing such interventions has been limited and systematic reviews show the need for systematically developed evidence-based interventions to support self-management of eczema.

3 & 4. WHAT (materials and procedures): Eczema Care Online for Young People is an online behavioural intervention for young people aged 13-25 years with mild to severe eczema. It aims to reduce eczema severity, targeting 5 key behaviours: (1) Increase their use of emollients to maintain skin hydration and prevent flare-ups; (2) Improve their use of TCS or TCIs through reactive applications of these treatments in response to flare-ups or, where appropriate, regular intermittent ("weekend") preventative applications of TCS/TCI if emollients are insufficient as maintenance therapy; (3) Improve their management of irritants and triggers; (4) Improve their emotional management; and (5) Reduce scratching. See Table S2 for an overview of the modules included in Eczema Care Online for Young People.

Table S2: Overview of the key intervention components of Eczema Care Online for Young People

Menu	Modules	Description
Home Page	N/A	• Signposting to key modules that young people haven't accessed yet<br>• Quick links to interactive content e.g. videos
Flare control creams	• Flare control cream video<br>• What are they?<br>• Are they safe?<br>• Common questions<br>• When and how do I use them?<br>• How can I find the right cream?<br>• Golden rules	• Evidence-based education and messages to increase motivation and competence to use topical corticosteroids (TCS), including; evidence of safety and support for skills development e.g. videos/photos showing use of TCS
Moisturising creams	• Moisturising creams video<br>• What are they and how do they help?<br>• Are they safe?<br>• Common Concerns<br>• When and how do I use them?<br>• How do I find the right cream?<br>• Take the two-week challenge<br>• Golden rules	• Evidence-based education and messages to increase motivation and competence to use emollients, including; evidence of safety and support for skills development e.g. videos/photos showing how to use emollients
What can make	• Living with eczema video	• Evidence-based education and messages to increase motivation and

eczema worse	• Bathing, showering, and washing clothes<br>• Cosmetics, make-up and shaving<br>• Diet and allergies<br>• Weather and holidays<br>• Swimming and physical activity	competence in identifying key eczema triggers and managing them, including practical tips
Living well with eczema	• Beat the itch<br>• Sleep<br>• Stress and eczema<br>• Studying and work<br>• Eczema and money	• Evidence-based education and messages to increase motivation and competence to manage some of the symptoms and psychosocial impacts of eczema e.g. itching and scratching, disturbed sleep, stress, including techniques to manage them
More about treatments	• Talking to health professionals<br>• Infections<br>• Topical Calcineurin Inhibitors<br>• Other treatments<br>• Other resources	• Evidence-based education and messages to increase motivation and competence to manage other aspects of treatments e.g. consulting with health professionals and other treatments

5. WHO PROVIDED: Eczema Care Online for Young People was provided direct to patients via their GP surgeries in this trial.

6. HOW: Eczema Care Online for Young People is entirely online, although users are encouraged to contact their GP, pharmacist or nurse, for example, to obtain or change their treatments, or to treat potential infections.

7. WHERE: Eczema Care Online for Young People is intended for any young person (aged 13-25) with mild to severe eczema. It can be implemented in primary or secondary care and can also be used stand-alone. The intervention is online and designed to be accessed using computers, tablets and phones via a web-browser. Although intended for use by young people there is no information that would be inappropriate for older age groups.

8. WHEN and HOW MUCH: For the randomised controlled trial study, young people were offered access to the intervention if their GP identified that they had eczema and had received a prescription for this in the previous 12 months. Once registered young people could use the online intervention as much or as little as they liked.

9. TAILORING: Young people had access to all modules on Eczema Care Online; however the website uses signposting based on four questions about the current state of their eczema. Users are then recommended either the module on emollients or flare control creams depending on whether it appears they are currently experiencing an eczema flare-up. Key modules can also be recommended, one or two at a time, on the intervention home page if they haven’t been viewed yet. These modules/menus are: 1) moisturising creams; 2) flare control creams, 3) bathing, showering and washing clothes; 4) beat the itch, 5) stress and eczema.

10. MODIFICATIONS: An additional email was circulated in response to COVID-19 that provided further advice about handwashing.

11. HOW WELL: The intervention has been evaluated in a randomised trial.

Table S3. Sensitivity analysis for parent/carer trial - POEM over 24 weeks (missing outcomes imputed)

Outcome	Mean score Usual care group	Mean score Online intervention group	Unadjusted mean difference in score (95% CI)	Adjusted[1] mean difference in score (95% CI)	Adjusted[2] mean difference in score (95% CI)
POEM over 24 weeks (missing outcomes imputed)	10.7	9.7	-1.0 (-2.1 to 0.1)	-1.1 (-1.9 to -0.2)	**-1.5 (-2.5** to **-0.5)**

[1]adjusted for stratification factors: baseline POEM, recruitment region, age

[2]adjusted for baseline POEM, recruitment region, age, gender, ethnicity, parental education, prior belief in the intervention and prior use of a website for eczema

Table S4. Sensitivity analysis for young people trial - POEM over 24 weeks (missing outcomes imputed)

	Usual care		Online intervention				
	N	Mean POEM score	N	Mean POEM score	Mean difference in POEM score (unadjusted)	Mean difference in POEM score (adjusted[1])	Mean difference in POEM score (adjusted[2])
Week 4	169	13.6	168	12.9	-0.7 (-2.0 to 0.6)	-0.6 (-1.6 to 0.5)	-0.2 (-1.4 to 1.0)
Week 8	169	13.2	168	12.0	-1.2 (-2.6 to 0.2)	-1.0 (-2.2 to 0.1)	-1.1 (-2.4 to 0.2)
Week 12	169	14.3	168	11.6	-2.7 (-4.2 to -1.3)	-2.5 (-3.7 to -1.3)	-2.7 (-4.1 to -1.3)
Week 16	169	14.4	168	10.9	-3.4 (-5.0 to -1.8)	-3.2 (-4.7 to -1.8)	-3.7 (-5.3 to -2.1)
Week 20	169	13.9	168	11.6	-2.3 (-3.9 to -0.6)	-2.1 (-3.6 to -0.6)	-2.1 (-3.7 to -0.5)
Week 24	169	13.9	168	11.7	-2.1 (-3.7 to -0.6)	-1.9 (-3.3 to -0.5)	-1.8 (-3.4 to -0.3)
POEM over 24 weeks		13.9		11.8	-2.1 (-3.2 to -0.9)	-1.9 (-2.8 to -0.9)	**-1.9 (-3.0** to **-0.8)**

[1]adjusted for stratification factors: baseline POEM, recruitment region and age

[2]adjusted for baseline POEM, recruitment region, age, gender, ethnicity, prior belief in the intervention and prior use of a website for eczema

Table S5. Exploratory subgroup analyses based on POEM at 24 weeks in trial for parents/carers

Subgroup		N	Mean difference in POEM score over 24 weeks (95 % CI)	Adjusted* interaction term (95% CI)	p-value for interaction term
Baseline POEM					
	Mild	53	0.8 (-0.8 to 2.4)		
	Moderate	211	-0.6 (-1.8 to 0.5)	-1.0 (-4.1 to 2.1)	0.53
	Severe	74	-4.0 (-6.7 to -1.4)	**-4.0 (-7.7** to **-0.2)**	0.04
Age group					
	0-4 years	216	-0.7 (-2.1 to 0.6)		
	5-12 years	122	-1.6 (-3.6 to 0.3)	-1.6 (-3.7 to 0.4)	0.12
Gender					
	Male	174	-0.9 (-2.5 to 0.6)		
	Female	163	-1.2 (-2.8 to 0.4)	1.4 (-0.5 to 3.3)	0.16
Emollient use					
	0-4 days	79	-0.3 (-2.4 to 1.9)		
	5-7 days	258	-1.1 (-2.4 to 0.2)	-0.5 (-2.7 to 1.7)	0.64
TCS use					
	0-4 days	261	-1.2 (-2.4 to 0.03)		
	5-7 days	75	-0.3 (-2.8 to 2.1)	0.7 (-1.6 to 3.0)	0.54
TCI use					
	0-4 days	295	-1.0 (-2.2 to 0.2)		
	5-7 days	10	-4.9 (-15.0 to 5.1)	2.5 (-4.5 to 9.5)	0.48
Prior belief					
	Low prior belief	20	-3.6 (-8.1 to 1.0)		
	Moderate prior belief	96	-0.1 (-1.9 to 1.8)	0.5 (-3.6 to 4.7)	0.81
	High prior belief	156	-1.8 (-3.8 to -0.02)	-0.7 (-4.7 to 3.3)	0.74
Use of other websites for eczema in last 6 months					
	No	261	-0.9 (-2.2 to 0.4)		
	Yes	72	-1.5 (-3.9 to 0.9)	-1.2 (-3.4 to 1.1)	0.32
Covid-19 pandemic**					
	Before	38	-0.8 (-4.4 to 2.8)		
	During	300	-1.1 (-2.3 to 0.1)	0.5 (-2.7 to 3.7)	0.77

*adjusted for baseline score, recruitment region, age, gender, ethnicity, parental education, prior belief in the intervention and prior use of a website for eczema

**Randomised before/after 11 March 2019

Significant results in **bold**

Table S6. Exploratory subgroup analyses based on POEM at 24 weeks[1] in trial for young people

Subgroup		N	Mean difference in POEM score at 24 weeks (95% CI)	Adjusted* interaction term (95% CI)	p-value for interaction term
Baseline POEM					
	Mild	18	-3.5 (-9.8 to 2.8)		
	Moderate	170	-2.0 (-3.9 to -0.05)	1.3 (-5.4 to 8.0)	0.70
	Severe	116	-2.3 (-4.8 to 0.2)	2.2 (-4.7 to 9.1)	0.54
Age group					
	13-17 years	108	-0.9 (-3.3 to 1.7)		
	18-25 years	196	-2.9 (-4.9 to -0.9)	-2.9 (-6.2 to 0.3)	0.08
Gender					
	Male	67	-1.5 (-4.5 to 1.5)		
	Female	237	-2.3 (-4.1 to -0.4)	-3.5 (-7.3 to 0.4)	0.08
Emollient use					
	0-4 days	107	-2.1 (-4.6 to 0.4)		
	5-7 days	194	-2.4 (-4.4 to -0.4)	0.9 (-2.3 to 4.2)	0.57
TCS use					
	0-4 days	240	-2.6 (-4.3 to -0.9)		
	5-7 days	60	-1.0 (-4.8 to 2.9)	-0.3 (-4.2 to 3.6)	0.88
TCI use					
	0-4 days	281	-2.1 (-3.7 to -0.5)		
	5-7 days	6	4.7 (-5.4 to 14.7)	2.3 (-8.7 to 13.4)	0.68
Prior belief					
	Low prior belief	23	1.7 (-4.6 to 8.0)		
	Moderate prior belief	101	-2.6 (-5.3 to 0.2)	-3.9 (-9.6 to 1.7)	0.17
	High prior belief	116	-1.6 (-4.4 to 0.7)	-4.8 (-10.3 to 0.7)	0.09
Use of other websites for eczema in last 6 months					
	No	251	-2.3 (-3.9 to -0.6)		
	Yes	45	-2.4 (-7.4 to 2.7)	-0.6 (-5.0 to 3.9)	0.81
Covid-19 pandemic**					
	Before	65	-0.8 (-4.4 to 2.9)		
	During	239	-2.6 (-4.3 to -0.9)	-1.2 (-5.0 to 2.7)	0.54

[1] Subgroup analysis carried out using only 24-week timepoint rather than repeated measures over all time points up to 24 weeks, due to treatment-time interaction

*adjusted for baseline POEM recruitment region, age, gender, ethnicity, prior belief in the intervention and prior use of a website for eczema

**Randomised before/after 11 March 2019

Significant results in **bold**
